# MicroRNA and messenger RNA profiling reveals new biomarkers and mechanisms for RDX induced neurotoxicity

**DOI:** 10.1186/1471-2164-15-S11-S1

**Published:** 2014-12-16

**Authors:** Youping Deng, Junmei Ai, Xin Guan, Zhaohui Wang, Bin Yan, Daqin Zhang, Chang Liu, Mitch S Wilbanks, Barbara Lynn Escalon, Sharon A Meyers, Mary Qu Yang, Edward J Perkins

**Affiliations:** 1 Wuhan University of Science and Technology, Wuhan, Hubei 430081, China; 2 Departments of Internal Medicine and Biochemistry, Rush University Medical Center, Chicago, IL 60612, USA; 3 Bristol Bay Native Corporation, Vicksburg, Mississippi 39180, USA; 4 Department of Biology, Hong Kong Baptist University, Kowloon, Hong Kong SAR, China Stem Cell & Regenerative Medicine Consortium, LKS Faculty of Medicine and Department of Physiology, The University of Hong Kong, Hong Kong SAR, China; 5 Center for Systems Biology, School of Mathematical Sciences, Soochow University, Suzhou, Jiangsu 215006, China; 6 United States Army Engineer Research and Development Center, 3909 Halls Ferry Road, Vicksburg, Mississippi 39180, USA; 7 School of Pharmacy, University of Louisiana at Monroe, Monroe, Louisiana, 71209, USA; 8 School of Computer Science and Technology, Wuhan University of Science and Technology, Wuhan, Hubei, PR China; 9 MidSouth Bioinformatics Center, Department of Information Science, George W. Donaghey College of Engineering and Information Technology, University of Arkansas at Little Rock, 2801 S. University Avenue, Little Rock, Arkansas, 72204, USA; 10 Joint Bioinformatics Graduate Program, University of Arkansas at Little Rock and University of Arkansas for Medical Sciences, Little Rock, Arkansas 72204, USA

## Abstract

**Background:**

RDX is a well-known pollutant to induce neurotoxicity. MicroRNAs (miRNA) and messenger RNA (mRNA) profiles are useful tools for toxicogenomics studies. It is worthy to integrate MiRNA and mRNA expression data to understand RDX-induced neurotoxicity.

**Results:**

Rats were treated with or without RDX for 48 h. Both miRNA and mRNA profiles were conducted using brain tissues. Nine miRNAs were significantly regulated by RDX. Of these, 6 and 3 miRNAs were up- and down-regulated respectively. The putative target genes of RDX-regulated miRNAs were highly nervous system function genes and pathways enriched. Fifteen differentially genes altered by RDX from mRNA profiles were the putative targets of regulated miRNAs. The induction of miR-71, miR-27ab, miR-98, and miR-135a expression by RDX, could reduce the expression of the genes POLE4, C5ORF13, SULF1 and ROCK2, and eventually induce neurotoxicity. Over-expression of miR-27ab, or reduction of the expression of unknown miRNAs by RDX, could up-regulate HMGCR expression and contribute to neurotoxicity. RDX regulated immune and inflammation response miRNAs and genes could contribute to RDX- induced neurotoxicity and other toxicities as well as animal defending reaction response to RDX exposure.

**Conclusions:**

Our results demonstrate that integrating miRNA and mRNA profiles is valuable to indentify novel biomarkers and molecular mechanisms for RDX-induced neurological disorder and neurotoxicity.

## Introduction

Hexahydro-1,3,5-trinitro-1,3,5-triazine (RDX), is a well known contaminant to territory, soil and ground water due to military and manufacturing activities. A series of studies have demonstrated that RDX can cause neurotoxicity including seizure in human and animals [1-4]. RDX can also induce immunotoxicity in rats [5-7].

While many effects of RDX exposure are known, the molecular mechanisms of RDX caused toxicity have not been well characterized. It appears that RDX binding to the GABA_A _receptor convulsant site maybe the primary mechanism of seizure induction by RDX and that reduction of GABAergic inhibitory transmission in the amygdala is involved in the generation of RDX-induced seizures[8-10]. But more mechanisms still needed to be studied such as epigenetic mechanisms.

Recently, It was [11] found RDX exposure could significantly alter a large number of miRNA expression in mouse brain and liver tissues with a 28 day long term exposure. MiRNAs are endogenous, small non-coding RNAs, usually 18-25 nucleotides long, have been found to play crucial roles in post-transcriptionally regulation of mRNA. MiRNAs have been found to involve in almost all fundamental important biological processes and diseases including neurological diseases and nervous system function [12]. MiRNAs carries out its function by specifically binding 3'UTR of mRNA to interrupt mRNA translation or cause degradation of transcripts [13-15]. Recent reports suggest that miRNA could play an opposite role by activating a gene expression at certain conditions [16,17].

We use rat as a model species to investigate the impact of RDX on both miRNA and mRNA expression in rat brain tissues with a sub-acute short term exposure (48 h). The objectives of the study are to see whether we could see an across species conserved miRNA expression between rat and mouse, find overlapped targets between the putative targets of regulated miRNAs and mRNA genes regulated by RDX, identify early expression altered miRNAs and genes as new markers for assessing RDX induced neurotoxicity, and further understand the molecular mechanisms of RDX caused neurotoxicity. Since that miRNAs are highly conserved between humans and rats, this study should improve our understanding of the molecular mechanisms of RDX induced neurological disorders and diseases.

There are still very few studies to use miRNA expression profiles for characterizing RDX caused toxicity. There is no report to integrate RDX altered miRNA and mRNA expression profiles.

## Materials and methods

### Chemical

RDX (purity > 99%) was obtained from Stan Caulder (Naval Surface Warfare Center, Indianhead, MD, USA).

### Animals and Treatment

Female Sprague-Dawley rats (175-225 grams) were from the in-house breeding colony (College of Pharmacy, University of Louisiana at Monroe [ULM] and treated in accordance with the *Guide for Use and Care of Animals *[18]. Breeders were from Harlan-Sprague Dawley in Madison, WI. Housing consisted of a 12 h light/dark cycle with *ad libitum *access to tap water and rodent chow (Harlan/Teklad 7012, Madison, WI). Rats were housed individually in polycarbonate cages on hardwood bedding (Sani-chips, Harlan/Tekland, Madison, WI) one week prior to treatment. Food was withdrawn the night before treatments, which were administered by gavage between 8 and 10 AM. Study protocols were preapproved by the Institutional Animal Care and Use Committee of the University of Louisiana at Monroe (Animal Welfare Assurance Number A3641-01).

Groups of rats were weighed and randomly assigned to treatment. Treatments were vehicle (5% v/v DMSO in corn oil), RDX (47 mg/kg). Rats were observed continuously for the first hour after dosing, hourly for 8 h and daily thereafter. Moribund rats were euthanized with CO_2_. At 48 hours after treatment, survivors were anesthetized with CO_2_. A portion of the brain was removed and flash frozen in liquid N_2 _and stored at -70º C for miRNA and mRNA microarray analyses.

### Total RNA extraction

Total RNA was extracted from about 30 mg of brain tissue. Tissues were homogenized in the lysis buffer with FAST Prep-24 from MP before using RNeasy kits (Qiagen). Total RNA concentrations were measured using NanoDrop® ND-1000 Spectrophotometer (NanoDrop technologies, Wilmington, DE, USA). The integrity and quality of total RNA was checked on an Agilent 2100 Bioanalyzer (Palo Alto, CA). The gel-like images generated by the Bioanalyzer show that total RNAs have two bands, represent 18S and 26S RNA of mammalian RNA. Nuclease-free water (Ambion) was used to elute total RNA.

#### MicroRNA extraction

We extracted total miRNA from each sample using the mirVana miRNA Isolation Kit (Ambion, Austin, TX) according to the manufacturer's instructions. Briefly, 0.03-0.05 g tissues were weighed and placed in a new 2-mL micro-centrifuge tube, followed by adding 300 μL lysis/binding buffer. Then, the tissues were thoroughly disrupted and homogenized using a Sonic Dismembrator (model 100, Fisher Scientific, Atlanta, GA). After homogenization, we added 30 μL miRNA homogenate additive to each tissue lysate, vortexed it for 10 sec, and then incubated it on ice for 10 min. After washing, we eluted the total RNAs using 100 μL elution buffer provided in the miRNA isolation kit. We performed all these operations on ice. The extracted RNA was quantified using a NanoDrop ND-100 spectrophotometer (NanoDrop Technologies, Wilmington, DE), aliquoted, and immediately stored at −80°C until analysis.

### MicroRNA microarray hybridization

The miRNA micro-array analysis was performed by LC Sciences (Houston, TX) as described previously [11]. Briefly, the assay started with approximately 6 μg total RNA. After the total RNAs were fractionated by size using a YM-100 Microcon centrifugal filter (Millipore, Billerica, MA), poly(A) tails were added to RNA sequences with lengths less than 300 nucleotides using poly(A) polymerase. Then, an oligonucleotide tag was ligated to the poly(A) tail for later fluorescent dye staining. RNA samples from brain extracts were hybridized overnight using two different tags on a μParaflo microfluidic chip using a microcirculation pump developed by Atactic Technologies (Houston, TX).

### mRNA microarray hybridization

mRNA microarray hybridization was done as described previously[19]. Briefly cDNA from 1 ug total RNA was synthesized, hybridized to arrays, and detected by secondary hybridization to Alexa647 and Cy3 dendrimer oligonucleotides using an Array900 detection kit per manufacturer's instructions (Genisphere, Hatfield, PA). cDNA was hybridized to 8 K Sigma/Compugen rat 70-mer oligonucleotide libraries arrayed on glass slides (Center for Applied Genomics, Newark, NJ http://www.cag.icph.org/).

### MiRNA microarray data analysis

MiRNA microarray was normalized by Lowess normalization algorithm using GeneSpring 10.0 (Agilent Technologies, Foster City, CA, USA). We first filtered substance according to flags. All substances that were at least 50% of samples in any of 2 out of 2 conditions (control group and RDX treatment group) have present calls according to the feature extraction software data will be remained. 215 substances were left after the flag filtering. We also judged a miRNA detectable using at least three criteria: *a*) signal intensity bigger than three times background standard deviation; *b*) spot coefficient of variance (CV) < 0.5, in which we calculated CV as (standard deviation)/(signal intensity); and *c*) at least two spots of four technical repeats in each chip and two of three biological replicates in either control or treatment group have signal greater than three times background standard deviation. We found that 178 miRNAs could be detected in either control or treatment group, and were also in the 215 miRNA list. Therefore, 178 detected miRNAs were employed to identify differentiated miRNAs in rat brain after exposure to RDX. Differentiated miRNAs were computed using un-paired T-test with a cut off p value <= 0.05 and fold change >=1.2 (corrected by Benjamini Hochberg at a FDR <= 0.05).

### MRNA microarray data analysis

MRNA microarray data analysis was done as we described previously [20,21]. Briefly, the data was normalized based on Lowess normalization method, Bayesian statistical analysis with 5% FDR was used to identify differential genes between two groups.

### MiRNA target prediction

Three algorithms were used to predict target genes of miRNAs. These three algorithms are miRBase [22], TargetScan [23] and PicTar [24].

### Reverse-transcription quantitative PCR (QRT-PCR)

We selected miRNAs with aberrant expression in microarray analysis and then validated them using qRT-PCR on an ABI7300 system (Applied Biosystems, Foster City, CA). We used TaqMan miRNA assays to detect and quantify mouse miRNAs using stem-loop RT-PCR according to the manufacturer's instructions.

### Gene functional analysis and network construction

Physiological process and pathway analyses were performed using the Ingenuity pathway analysis (IPA) tool. A physiological process or a pathway with an enrichment p value <= 0.05 was considered to be significant (Ingenuity Systems, Inc., Redwood City, CA). Gene networks were constructed based on the IPA tool. A score was assigned to a network according to the fit of the original set of significant genes. This score reflects the negative logarithm of the p value that indicates the likelihood of the focus genes in a network being found together due to random chance [25].

## Results

### Regulation of miRNA expression in the brain tissues of rats exposed to RDX

**Figure 1:**
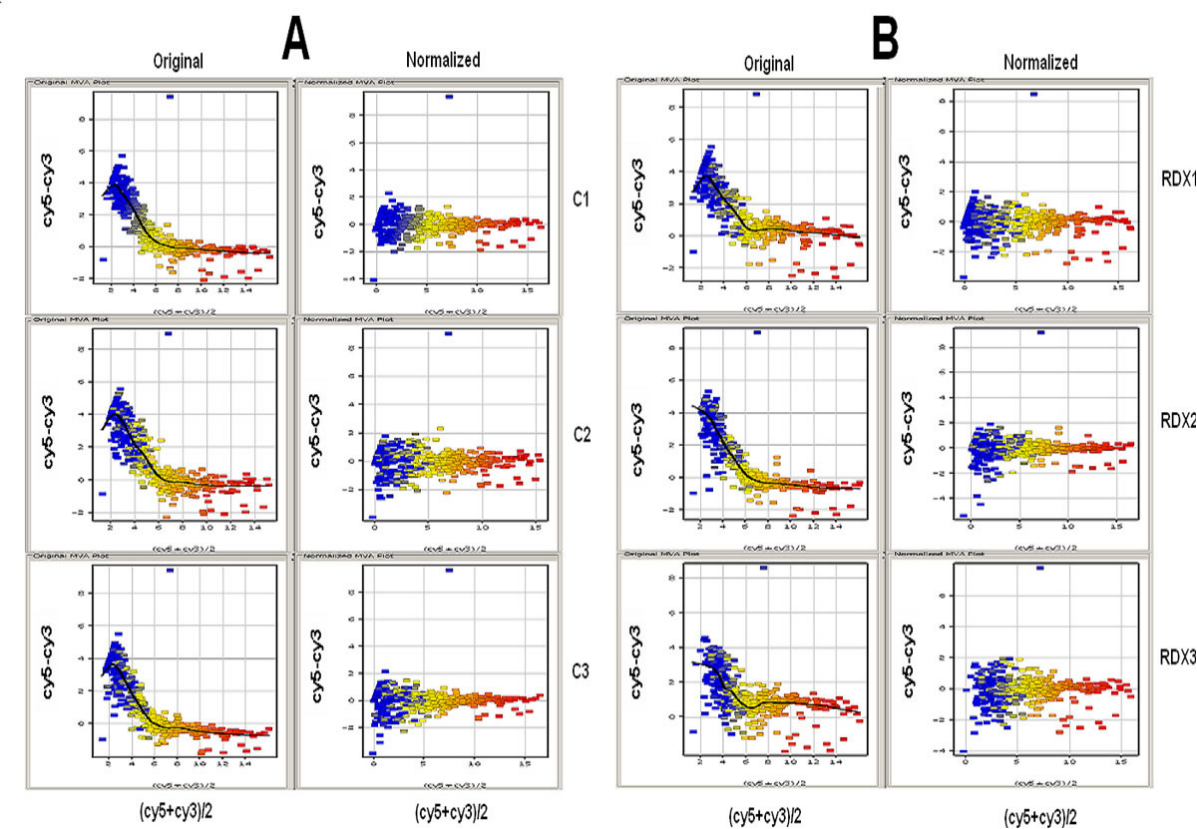
**Impact of normalization on the data distribution of control (A) and RDX treated (B) samples**. Lowess normalization algorithm was employed and bias skewed data distribution was observed in both control (A) and RDX treated (B) original samples, but was removed in the normalized samples.

To determine whether the expression profile of miRNAs changes in response or RDX, we analyzed global expression of mature miRNAs in the brain tissues of rats treated with RDX. Rats were treated with RDX at a dose (47 mg/kg) for 48 h. Three control and treatment animals were used. Brain tissues from treated and control rats were used for miRNA profiling. There were 344 rat miRNAs and 50 control molecules, and each miRNA was represented by 4 technical replicated spots on each chip. The Lowess normalization was employed to remove the systematic error of the experiments. Regardless control (Figure 1A) or RDX (Figure 1B) treated samples, the original data showed a clear bias skewed distribution. After the normalization, the bias distribution was removed in both control (Figure 1A) and RDX (Figure 1B) treated samples. Nine miRNAs were differentially regulated by RDX with a cut off p values (<= 0.05) and fold change (>= 1.2) (Table 1). Of these 9 miRNAs, 6 miRNAs including miR-98, miR-27b, miR135a, miR-7a, miR-674-5p and miR-27a were significantly up-regulated by RDX. The expression of miR-320, miR-129* and miR-342-3p was significantly down-regulated by RDX.

**Table 1:** Significantly regulated miRNAs in rat brain after exposure to RDX.

MiRNA name	p-value	Fold change	Regulation
miR-98	0.006792	1.715814	up
miR-27b	0.029563	1.59366	up
miR-135a	0.019873	1.542563	up
miR-7a	0.048486	1.349329	up
miR-674-5p	0.009661	1.263634	up
miR-27a	0.041365	1.227918	up
miR-320	0.04012	-1.582334	down
miR-129*	0.039644	-1.476831	down
miR-342-3p	0.026962	-1.28396	down

**Figure 2:**
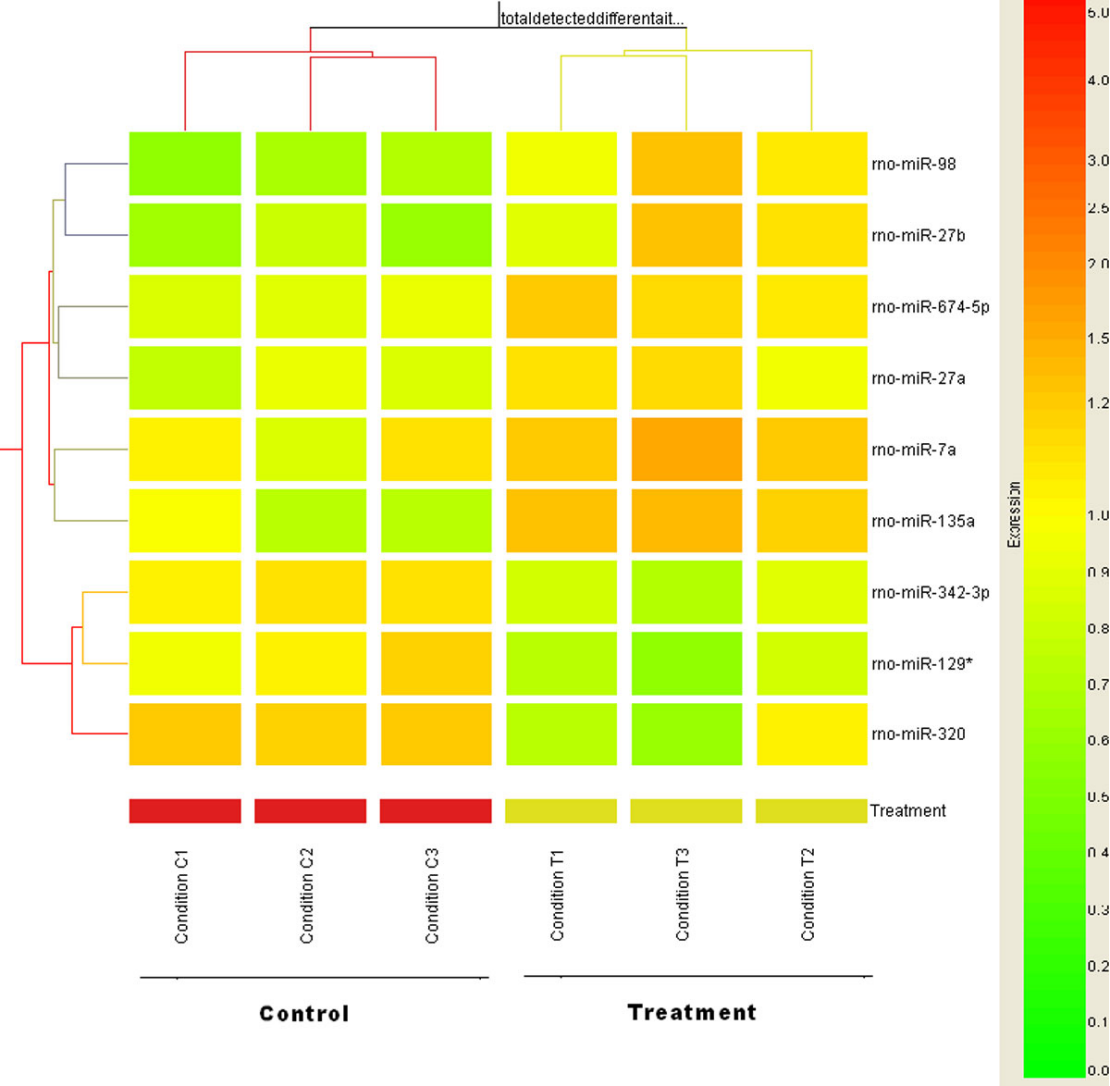
**Hierarchical clustering of differentially expressed miRNAs induced by RDX**. Nine differentially expressed miRNAs (horizontal axis) were used for a Two-Way hierarchical clustering across all the control and RDX treated samples (vertical axis). A Pearson correlation algorithm was applied to calculate the distances between transcripts or between conditions. The relative level of gene expression is indicated by the color scale at the right side.

Using these 9 miRNAs, we performed a two-way hierarchical clustering analysis. The samples were divided into two clusters: three control samples were in a one cluster and three RDX treated sample were in the other cluster. The 9 miRNAs were also grouped into two clusters: 6 up-regulated miRNAs in a cluster and 3 down-regulated miRNAs in the other cluster (Figure 2).

### Computational predictions of the putative targets of regulated miRNAs

**Figure 3:**
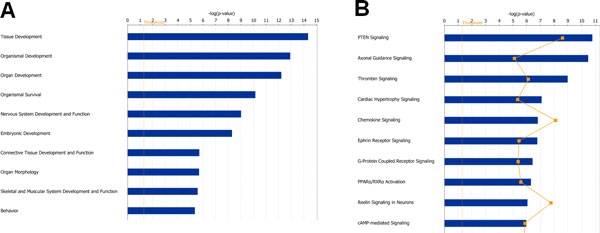
**Most significantly physiological processes (A) and canonical pathways (B) of the putative target genes of RDX regulated miRNAs**. Top ten physiological processes (A) and pathways (B) were selected to present. The putative target genes of RDX regulated miRNAs in rat brain tissues were used to run the Ingenuity pathway tool. The bigger the -log(p-value) of a pathway is, the more significantly the physiological process or pathway is regulated. The threshold lines represent a p value with 0.05.

In order to identify the putative targets or regulated miRNAs, we selected three most popular computational algorithms: miRBase, PicTar and TargetScan. We chose target genes based on a target gene that was recognized by at least two algorithms. In addition, we also counted target genes that were targeted by two or more of these regulated miRNAs. A total of 1589 target genes were predicted. Based on the IPA tool, we analyzed the physiological development and function categories of these target genes. Top significant enriched development and function terms were presented in Figure 3A. We could observe that the nervous system development and function category in the top list, which included 256 target genes (Additional file 1) of the miRNAs. Another function category behavior which is also associated with brain function was in the top list, which contained 86 genes.

Figure 3B exhibited top 10 significant canonical pathways enriched in the 1589 target genes. Among them, two canonical pathways axonal guidance signaling and reelin signaling in neurons were clearly involved in nervous system function. Seventy six target genes (Additional file 2) fell into the axonal signaling pathway which is related to neurotransmitters and other nervous System Signaling. Twenty-two genes (Additional file 2) play a role in reelin signaling in neurons pathway.

**Figure 4:**
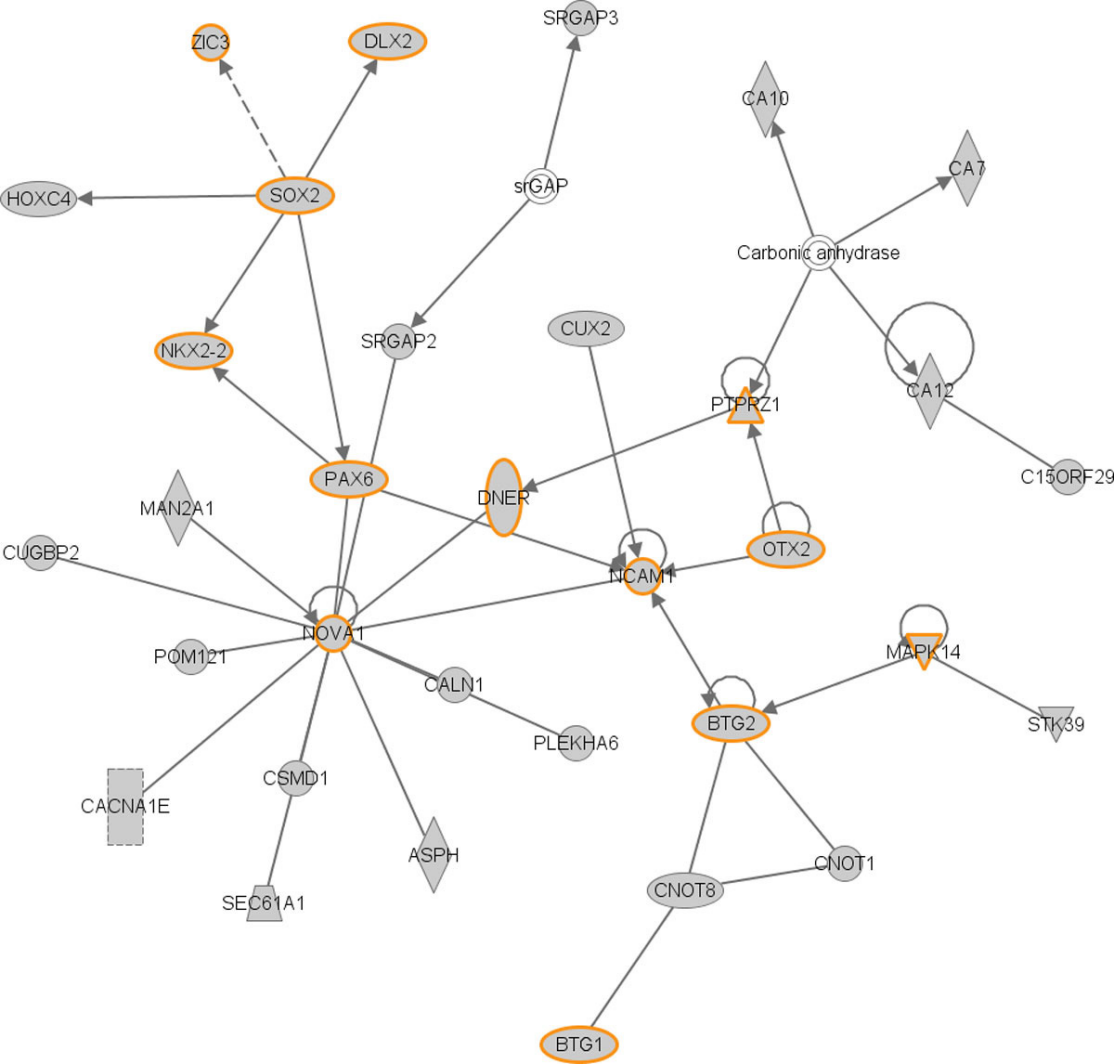
**Gene network of the putative target genes of RDX regulated miRNAs**. The putative target genes of RDX induced differentially expressed miRNAS were used to run the IPA tool for gene network analysis. The orange highlighted genes are involved in nervous system development function. The networks score described in Materials and Methods for the network is 39. The solid lines connecting molecules here represents a direct relation and dotted lines an indirect relation.

In order to find out the interaction between target genes, we generated common networks using the IPA software. The target genes of the regulated miRNAs by RDX were uploaded into the IPA software tool. Networks formed by these genes were then mathematically generated according to their connectivity. Figure 4 illustrates the graphical representation of themolecular relationships between genes, which is one of gene networks developed by IPA with the highest score (39). The network is associated with cellular development, nervous system development and function, organism development. Several transcription factors such as DLX2, SOX2, NKX2-2, PAX6, OTX2, BTG1 and BTG2, and protein kinases such as PTRZ1, MAPK14 were found to be involved in the network especially related to nervous system development and function. This network also exhibited that many of these genes were regulated by each other either directly or indirectly.

### Comparison of miRNA target genes and mRNA expression profile

The overlap between putative targets of miRNAs and the expression of mRNAs offers the further information on the biological processes and specific miRNA regulated gene networks. To reach the goal, we also conducted mRNA microarray analysis. Rats were treated with or without RDX at (47 mg/kg) for 48 h, subsequently the mRNA level of different genes in the brain tissues were monitored by cDNA micorarray techniques with approximately 8,000 probes. Using a Bayesian statistical analysis with 5% FDR, a total of 123 genes were significantly regulated by RDX. Of these genes, 60 genes were up-regulated (Additional file 3) and 63 genes were down-regulated (Additional file 4). In total, 15 (12.2%) of these regulated genes were putative targets of miRNAs regulated by RDX. Ten and 5 of these overlapped genes were down-regulated and up-regulated by RDX respectively (Table 2). This relationship maybe inverse, for instance, down-regulated miRNAs and over-expressed targets and up-regulated miRNAs and down-regulated targets. As shown in table 3 the targets of miR-135a, miR-98, and most of the targets of miR-7a have an inverse relationship with their responsive miRNAs. However, some positive relationship also occurred here. MiR-320, rno-miR-129* and their targets were both repressed by RDX. Rno-miR-647-5p and its target gene VGF were both induced by RDX (Table 3).

**Table 2:** Overlapped genes between putative target genes of miRNAs and differential mRNA genes regulated by RDX.

Symbol	Entrez Gene Name	Regulation
HMGCR	3-hydroxy-3-methylglutaryl-CoA reductase	Up
LITAF	lipopolysaccharide-induced TNF factor	Up
NPTX2	neuronal pentraxin II	Up
SLC38A2	solute carrier family 38, member 2	Up
VGF	VGF nerve growth factor inducible	Up
BANP	BTG3 associated nuclear protein	Down
C5ORF13	chromosome 5 open reading frame 13	Down
CALD1	caldesmon 1	Down
CEP350	centrosomal protein 350 kDa	Down
FAM82A2	family with sequence similarity 82, member A2	Down
KIAA1033	KIAA1033	Down
POLE4	polymerase (DNA-directed), epsilon 4 (p12 subunit)	Down
ROCK2	Rho-associated, coiled-coil containing protein kinase 2	Down
SLC35E4	solute carrier family 35, member E4	Down
SULF1	sulfatase 1	Down

**Table 3:** The relationship between miRNAs and their target genes regulated by RDX.

MiRNAs		Target Genes	
**Names**	**Regulation**	**Up**	**Down**

miR-135a	Up		KIAA1033, CEP350, ROCK2
miR-320	Down		BANP, CALD1, POLE4
miR-98	Up		SULF1
miR-129*	Down		BANP, FAM82A2
miR-27ab	Up	HMGCR, LITAF, NPTX2	CALD1, C5orf13, KIAA1033
miR-342-3P	Down	VGF	KIAA1033
miR-7a	Up	SLC38A2	POLE4, SLC35E4, C5orf13
miR-674-5p	Up	VGF	

Biological functional analysis revealed that more than half (8) of the overlapped genes are involved in neurological diseases and nervous system function (Table 4). These genes included HMGCR, BANP, C5ORF13, ROCK2, LITAF, POLE4, SULF1 and VGF.

**Table 4:** Over half (8) of the overlapped target genes are involved in neurological disease and nervous system function.

Category	Function Annotation	P-value	Molecules	# Molecules
Neurological Disease	granular cell tumor	1.98E-03	HMGCR	1
Neurological Disease	Huntington's disease	2.04E-03	BANP, C5ORF13, HMGCR, ROCK2	4
Neurological Disease	subarachnoid hemorrhage	2.98E-03	HMGCR	1
Neurological Disease	neurofibromatosis	6.93E-03	HMGCR	1
Neurological Disease	neurological disorder	1.11E-02	BANP, C5ORF13, HMGCR, LITAF, POLE4, ROCK2	6
Neurological Disease	meningitis	1.68E-02	POLE4	1
Neurological Disease	Charcot-Marie-Tooth disease	2.16E-02	LITAF	1
Neurological Disease	medulloblastoma	3.90E-02	POLE4	1
Neurological Disease	migration of brain cancer cell lines	4.09E-02	C5ORF13	1
Neurological Disease	stroke	4.19E-02	HMGCR	1
Neurological Disease	neuropathy	4.77E-02	HMGCR, LITAF	2
Nervous System Development and Function	morphology of dendritic spines	9.89E-03	ROCK2	1
Nervous System Development and Function	sprouting of neurites	2.07E-02	SULF1	1
Nervous System Development and Function	differentiation of neurons	2.42E-02	C5ORF13, VGF	2
Nervous System Development and Function	retraction of neurites	4.57E-02	ROCK2	1

### Comparative pathway and network analysis

**Figure 5:**
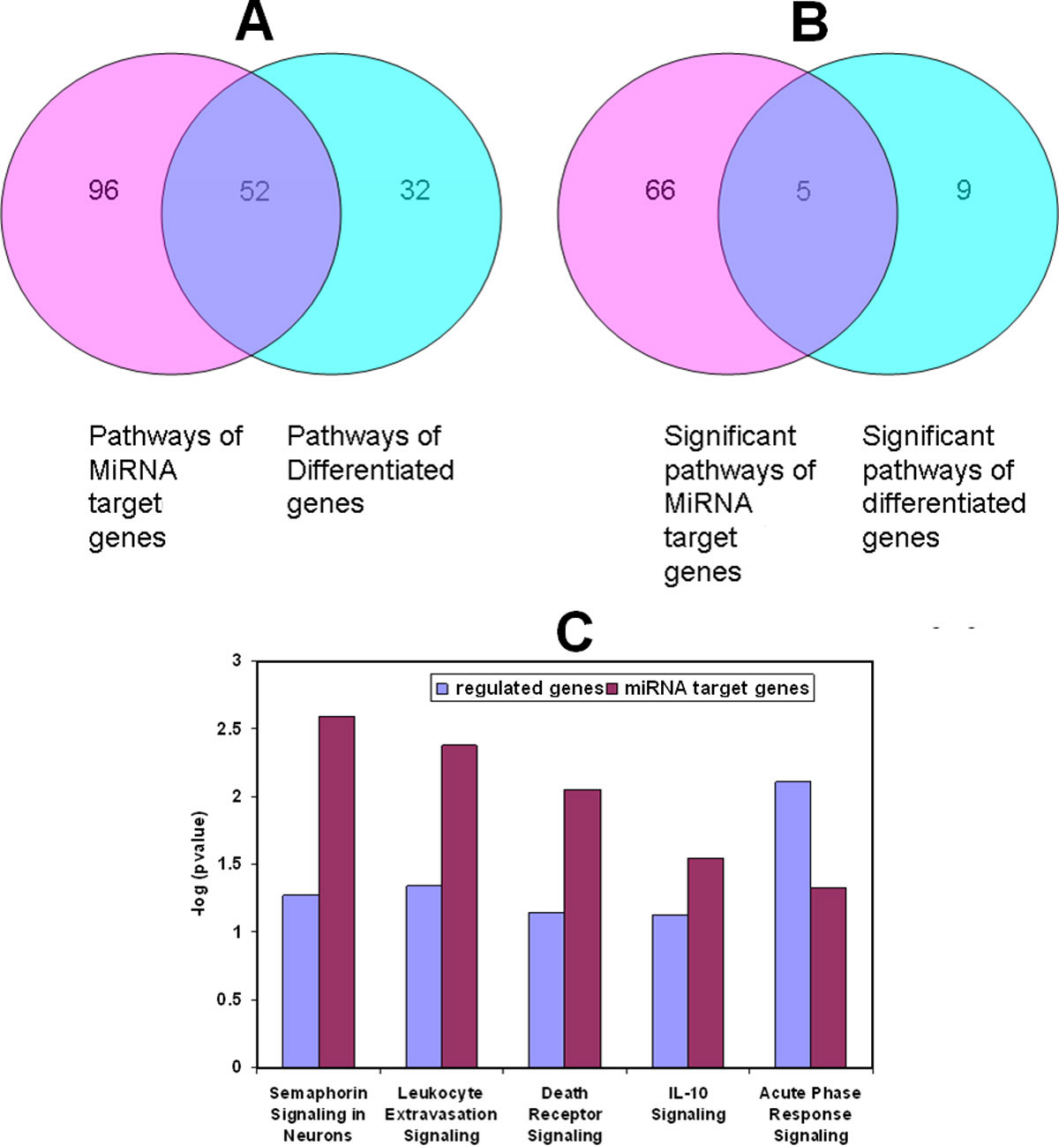
**Comparison of canonical pathways based on the putative target genes of RDX regulated miRNAs and differentiated mRNA genes**. Overlapped all mapped pathways (A) or significant pathways (B) based on the putative target genes of RDX regulated miRNAs and differentiated mRNA genes, are presented in the Venn diagrams. The IPA tool was used for the pathway analyses. A pathway enrichment p value less than 0.05 was considered as significant. The overlapped significant pathway names and their significance are depicted too (C).

Besides identifying common genes between putative target genes of regulated miRNAs and mRNA expression, we further compared common pathways. Pathway analyses were performed using the IPA tool. Two gene lists: putative target genes of regulated miRNAs and differential genes from the mRNA microarray profiles were uploaded into the IPA tool for pathway mapping. Based on all mapped pathways, 52 pathways were shared by these two gene lists (Figure 5A), which is 61.9% of the 84 mapped pathways of the mRNA genes regulated by RDX. Using only significantly mapped pathways (p value <= 0.05), 5 pathways were overlapped (Figure 5B), which is 55.6% of the 14 significant pathways of the mRNA genes affected by RDX. The five common significant pathways are shown in Figure 5C. Clearly we could observe one significant pathway seaphorin signaling in neurons is involved in neural system function.

**Figure 6:**
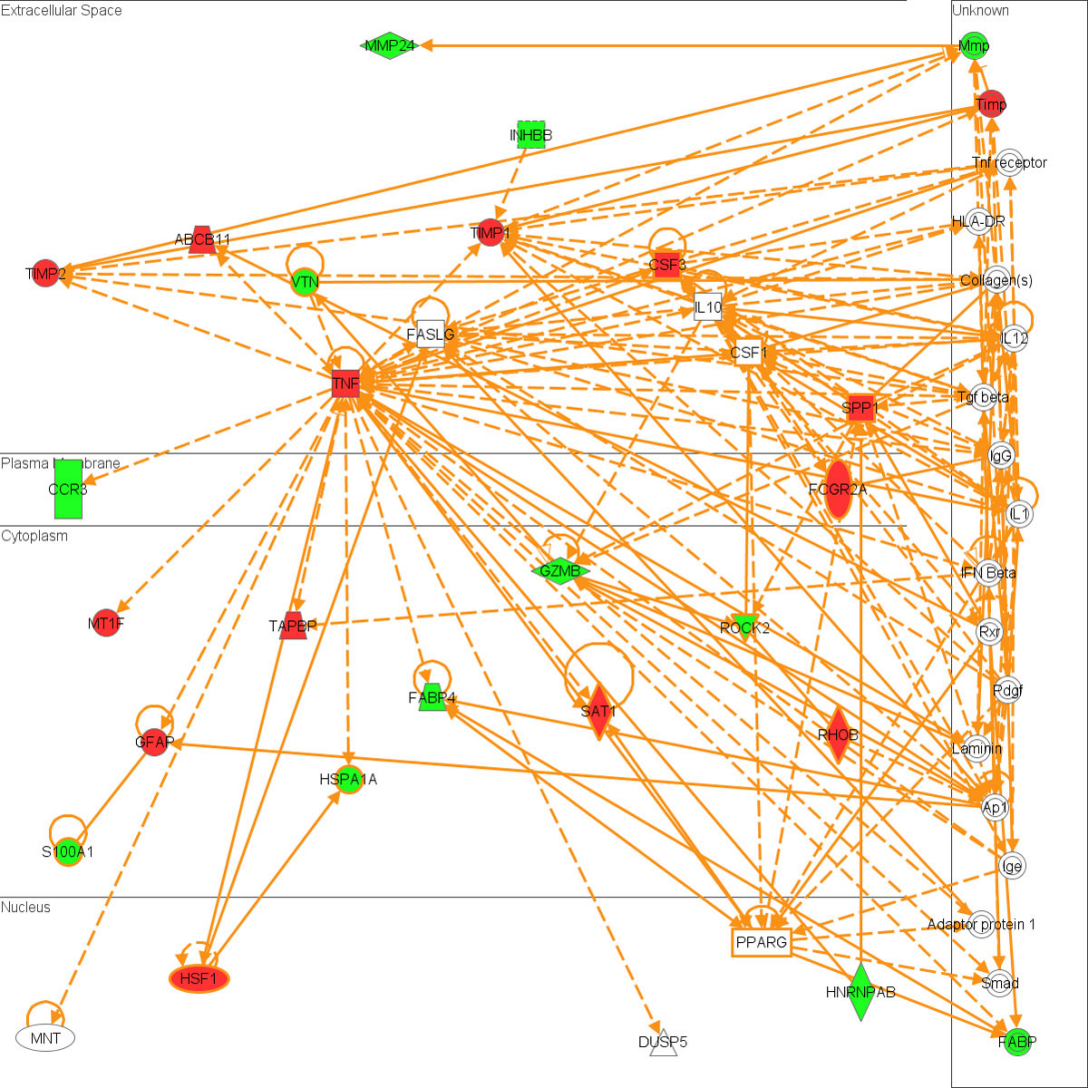
**Gene network using immune and inflammation response genes from both the putative target genes of RDX regulated miRNAs and differentiated mRNA genes**. The network indicates that RDX could first modulate miRNA expression and then trigger an immune and inflammation gene network by indirectly regulating some immune and inflammation response genes. The network was built using the IPA tool. Nodes colored in red or green denote up-regulated and down-regulated genes respectively.

Interestingly, a large group of genes regulated by RDX through the mRNA profiles were immune and inflammatory response genes. For example, the most significantly regulated pathway for the regulated mRNA gene list (Figure 5C) was acute phase response signaling. But most of these immune and inflammatory response genes were not significantly represented in the putative target genes of miRNAs regulated by RDX. MiRNAs may indirectly regulate these mRNA genes through directly regulating other genes. Using both immune and inflammatory response genes from both putative target genes of regulated miRNAs and regulated mRNA genes, a gene network was constructed (Figure 6). We could obviously see the gene interaction between these two sources of genes. For example, the putative target gene IL10 could regulate mRNA genes TIMP1, GZMB and TNF. TNF could also regulate putative target genes MNT and DUSP5 to carry out immune and inflammatory functions.

### Verification of miRNA microarray responses using real time QRT-PCR

**Figure 7:**
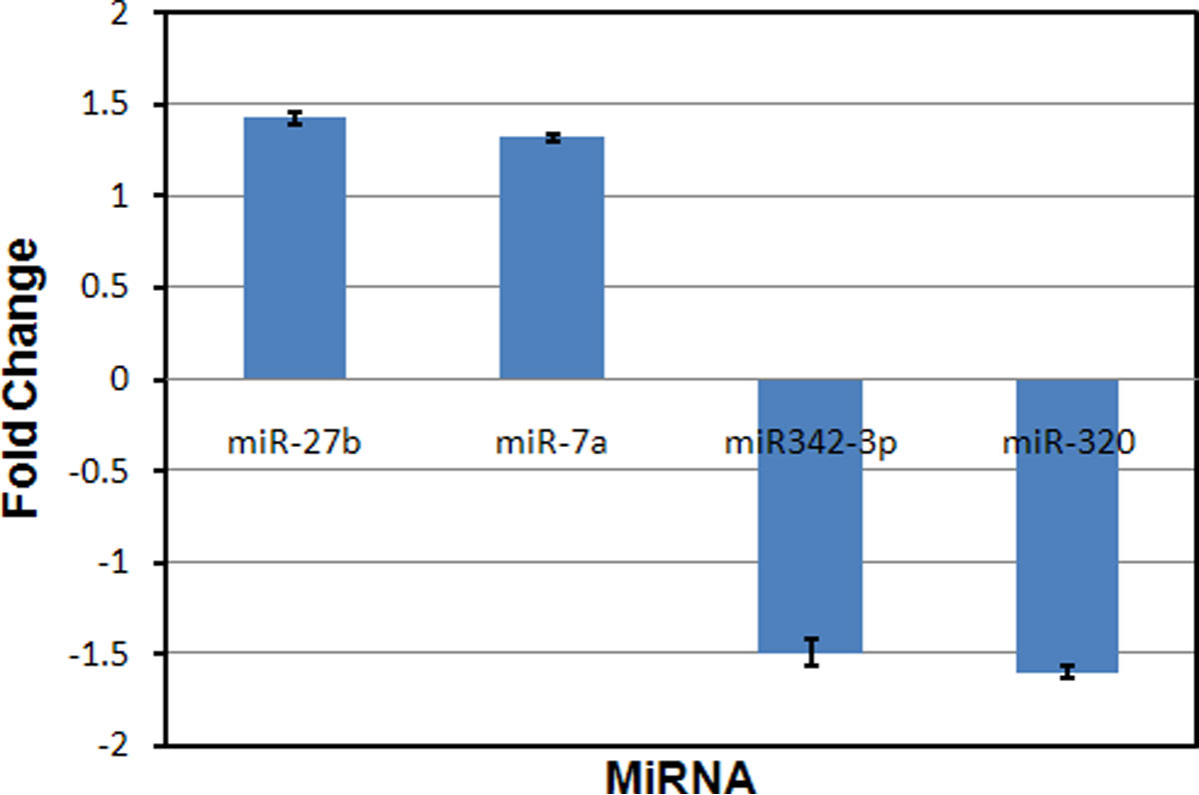
**Verification of miRNA microarray responses using real time QRT-PCR**. The fold changes represent the expression values of the miRNAs of the RDX-treated versus control samples. Values refer to the mean ± SD of three independent samples, each run in triplicate.

To verify miRNA microarray results, we selected 4 miRNAs to perform real time quantitative PCR (QRT-PCR) for the RDX exposed brain samples and their controls. As illustrated in Figure 7, miR-27b and miR-7a were up-regulated whereas miR-343-3p and miR-320 were down-regulated by RDX. The QRT-PCR results were consistent with the miRNA microarray results (Table 1).

## Discussion

### MiRNA alteration in response to RDX exposure

This is the first report that comprehensively analyzes the rat brain miRNA expression profile in response to RDX exposure. We found that 9 miRNAs whose expression was altered in rat brain tissues by RDX. Among them, 6 miRNAs were up-regulated and 3 miRNAs were down-regulated. Two miRNAs miR-98 and miR-7a were induced in rat brain tissues after exposure to RDX, and they were also up-regulated in mouse brain tissues treated with RDX [11]. MiR-27b and miR-320 were up-regulated and down-regulated respectively in our study, but not significantly changed in mouse brain tissue. However they were altered in mouse liver tissues with the same direction as rat brain tissues by RDX. Some of our regulated miRNA are not shown in Zhang's regulated miRNA lists, and also RDX induced much more miRNAs in mouse brain than our case. This should not be hard to explain, because Zhang's study is a long term exposure (28 days), we only used 2 day short exposure. It may also have dose and species specific responses.

Some of these 9 regulated miRNAs have been shown to participate in neurological diseases and neural system function. For instance, miR-7a was reported to be involved in glioblastoma [26] and Parkinson's disease [27]. MiR-27a and MiR-129 were shown to play a role in autism spectrum disorder (ASD) [28]. MiR-320 was observed to be involved in neurodegenration [12] and retinoblastoma [29]. MiR-342-3p has been found to play a role in neurodegenration [12] and prion disease [30]. Our results indicate that RDX could induce neurological diseases and neurotoxicity through regulating these miRNAs.

### The putative targets of RDX regulated miRNAs involved in neurotoxicity

Existing studies have exhibited that RDX exposure induced adverse central nervous system (CNS) syndromes such as convulsion, epileptic seizure, and loss of reflexes in human and experimental animals [1,5-7]. However, how RDX causes neurotoxicity at molecular level is not well known. Through the functional analyses of these target genes, we found that multiple evidences to support that RDX affects neural system development and neurological pathways. Nervous system development and function is in the top significant physiological system development and function category (Figure 3A). Axonal guidance signaling pathway is in the top 2 significantly pathways enriched in the canonical pathway analysis (Figure 3B). The pathway plays a crucial role in neuronal connections that are formed by the extension of axons, which migrate to reach their synaptic targets. The axonal growth cone, located at the axon leading edge, contains receptors that sense attractive and repulsive guidance cues, which help navigate the axon to its final destination [31-35].

Another important pathway in the top significant pathway list (Figure 3B) is reelin signaling in neurons. Reelin is a large extracellular glycoprotein involved in the development of architectonic patterns, and Cajal-Retzius cells of the human embryonic marginal zone are the primary site of synthesis for reelin. In the hippocampus, reelin also regulates the growth and/or distribution of afferent entorhinal and commissural axons. The genes in the pathway provide molecular mechanisms that control brain development and, potentially, the pathogenesis of neurodegenerative disorders [36-38]. Moreover, the most significant gene network based on the putative target genes is involved in neural system function and development (Figure 4). Regulating the gene network and the pathways could partially explain the molecular mechanism for RDX induced neurotoxicity.

### Overlapped genes between putative targets of regulated miRNAs and differential mRNA genes revealing new markers for RDX induced neurotoxicity

We found 15 overlapped genes, which are about 12% of differential mRNA genes (123 genes). The number is not big and the percentage is similar to other studies. For instance, the overlapped target genes was 12% in prion induced miRNA and mRNA profiles [12]. The Several reasons could account for the small portion of overlapping. First, the rat mRNA array is not the whole genome array, only about one third of the genome (8000 probes) is used, but the prediction of miRNA targets is based on the whole genome. Second, the prediction is static, which means the targets are predicted based on any condition, but the mRNA genes are differentially expressed at a certain condition. Third, the predictive putative targets may contain many false positives. Fourth, technical limitation for both miRNA and mRNA arrays may also leave out potential overlapped genes.

**Figure 8:**
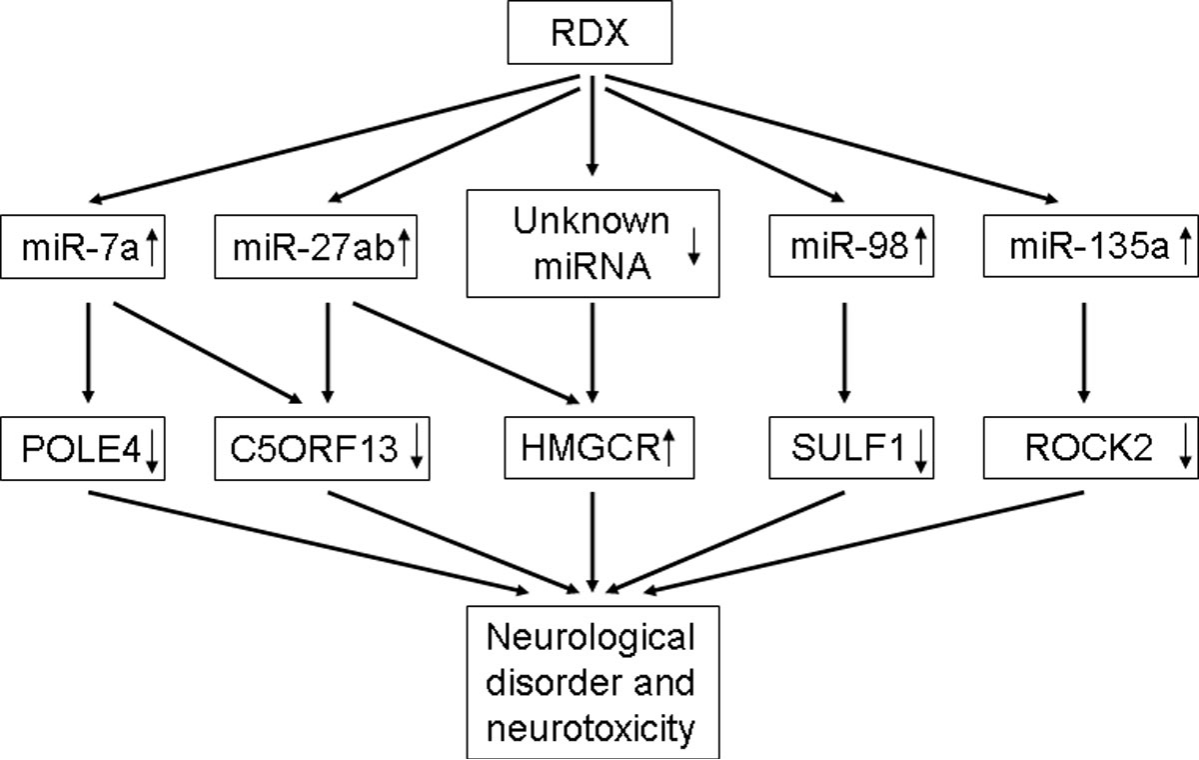
**A hypothetical model to explain the molecular mechanisms of RDX-induced neurological disorder and neurotoxicity**. RDX could first up-regulate the expression of miR-71, miR-27ab, miR-98, and miR-135a, then reduce the expression of the gens POLE4, C5ORF13, SULF1 and ROCK2, and eventually induce neurotoxicity. Over-expression of miR-27ab, or reduction of the expression of unknown miRNAs by RDX, could up-regulate HMGCR expression to cause neurotoxicity too.

We did see an inverse relationship between the regulated miRNA expression and their target mRNA genes (Table 3). However, we also observed a positive relationship between the expression of the miRNA and their target genes (Table 3). Normally the enhancement of miRNA expression inhibits target mRNA expression, but it could also facilitate target mRNA expression [16,17]. Over half of the overlapped target genes are involved in neurological disease and nervous system function (Table 4). If we only consider the inverse correlation between miRNAs and their target genes, particularly those target genes involved in neurological function that are repressed due to the over-expression of the regulated miRNAs, several target genes stand out and are SULF1, C5ORF13, ROCK2, POLE4. SULF1 is necessary for sprouting of neurite, and is involved in neurological disorder, ischemic stroke and bipolar disorder [39]. C5ORF13 plays a role in accelerating nerve regeneration of the axtomized facial nerve and has been found to involve in multiple neurological diseases [40]. ROCK2, a Rho-associated, coiled-coil containing protein kinase, is involved in reaction of neurites [41]. POLE4 also contributes to multiple neurological diseases (Table 4). Here we also want to point out one target gene HMGCR whose expression is increased by RDX. It has been found to its over expression could induce stroke and seizure [42,43]. The up-regulation of HMGCR could be one reason for RDX induced stroke and seizure. But we only found up-regulated miRNAs miR-27a and miR-27b that could target HMGCR. There may be unknown miRNAs that target HMGCR and are down-regulated by RDX. Therefore, based on both miRNA and mRNA profiles, we come out a hypothetic model that could partially explain RDX trigged neurological disorder and neurotoxicity. RDX could first induce the expression of miR-71, miR-27ab, miR-98, and miR-135a, then reduce the expression of POLE4, C5ORF13, SULF1 and ROCK2, and finally induce neurotoxicity. RDX induced miR-27ab over-expression, or reduce the expression of unknown miRNAs, could consequently up-regulate HMGCR expression to cause neurotoxicity too (Figure 8). The miRNAs and genes in the model could be new biomarkers for RDX induced neurotoxicity, which could work together with RDX binding to GABAa receptor to explain RDX caused brain damage.

### MiRNAs mediated gene network of immune and inflammation response genes regulated by RDX

Immune and inflammation response genes are highly enriched in differentially expressed mRNAs after exposure to RDX, but not in the putative target genes of regulated miRNAs. Interestingly, the immune and inflammation response genes connected network (Figure 6) indicates that RDX could first modulate miRNA expression and then trigger an immune and inflammation gene network. For instance, miR-98 could target IL10, which then up-regulate TNF, which induces the expression of the transcription factor HSF1 (Figure 6), to carry out immune functions. RDX regulated immune response could contribute to RDX induced neurotoxicity and other toxicities as well as animal defending reaction response to RDX exposure.

## Conclusions

Our results demonstrate that integrating miRNA and mRNA profiles is valuable to indentify novel biomarkers and molecular mechanisms for RDX-induced neurological disorder and neurotoxicity. RDX could first induce the expression of miR-71, miR-27ab, miR-98, and miR-135a, then reduce the expression of POLE4, C5ORF13, SULF1 and ROCK2, and finally induce neurotoxicity, which provides an alternative mechanism to explain RDX induced neurotoxicity.

## Competing interests

The authors declare that they have no competing interests.

## Authors' contributions

YD and EJP designed and coordinated the study. YD, JA, MQY, YB, DZ, CL and ZW analyzed miRNA and mRNA array data. XG, MSW, BLE and SAM performed the experiments. YD drafted the manuscript. YD and EJP revised and finalized the manuscript which was read and approved by all authors.

## Supplementary Material

Additional file 1 Target genes related to nervous development. Click here for file

Additional file 2 Canonical pathways enriched in the miRNA target genes. Click here for file

Additional file 3 Up-regulated genes by RDX. Click here for file

Additional file 4 Down-regulated genes by RDX. Click here for file
